# Inhibition by fluoxetine of LH-stimulated cyclic AMP synthesis in tumor Leydig cells partly involves AMPK activation

**DOI:** 10.1371/journal.pone.0217519

**Published:** 2019-06-04

**Authors:** Thi Mong Diep Nguyen, Danièle Klett, Laura Filliatreau, Yves Combarnous

**Affiliations:** 1 INRA, CNRS, Physiologie de la Reproduction et des Comportements Nouzilly, France; 2 Faculty of Biology-Agricultural Engineering, Quy Nhon University, Quy Nhon, Viet Nam; Georgia State University, UNITED STATES

## Abstract

Fluoxetine (FLX), a widely used antidepressant primarily acting as a selective serotonin reuptake inhibitor (SSRI), has been shown to exhibit other mechanisms of action in various cell types. Consequently, it might have unexpected adverse effects not related to its intended use, possibly in the endocrine regulation of reproduction. We show in the present report that after a 1-hour preincubation of MLTC-1 Leydig cells with FLX, the intracellular cyclic adenosine monophosphate (cAMP) responses to Luteinizing Hormone (LH) and forskolin (FSK) are reduced through AMPK-dependent and -independent pathways respectively. FLX at low concentrations (12.5μM and 25μM) induced this inhibition without triggering AMPK phosphorylation, while higher FLX concentrations (50μM and 100μM) induced AMPK phosphorylation and lowered ATP concentration similarly to Metformin. Pretreatment with the specific AMPK inhibitor Compound C (CpdC), significantly diminished the inhibition of cAMP synthesis caused by high concentration of FLX. Moreover, as expected FLX also caused a decline of steroidogenesis which is under the control of cAMP. Taken together, these findings demonstrate that the inhibition of cAMP synthesis by FLX is dose-dependent and occurs in MLTC-1 cells through two mechanisms, AMPK-independent and AMPK-dependent, at low and high concentrations, respectively. FLX also inhibited hormone-induced steroidogenesis in MLTC-1 cells and mouse testicular Leydig cells, suggesting similar mechanisms in both cell types.

## Introduction

Fluoxetine (FLX), the active molecule in Prozac, is a drug used to fight symptoms of conditions such as major depression, obsessive-compulsive disorder, bulimia nervosa and panic disorder, dysautonomia, postpartum depression, premature ejaculation, fibromyalgia or trichotillomania [1–2]. It primarily acts as a selective serotonin reuptake inhibitor [3], but also inhibits various ion channels [4–8] as well as the respiratory chain in mitochondria [9]. Consequently, it is expected to lower ATP production and thus to stimulate 5’-AMP activated protein kinase (AMPK) activity.

AMPK is a key regulator of cellular energy homeostasis involved in the regulation of fatty acid, cholesterol synthesis [10] and many other anabolic pathways [11–12] and its expression in gonads has been clearly evidenced [3–8]. Since AMPK is present in ovaries (granulosa, theca, oocytes and corpora luteal cells) and testes (Sertoli, Leydig and germinal cells) of many species [13–16], its potential interferences with gonadal cell responses to gonadotropins must be taken into account.

The gonadotropin Luteinizing Hormone (LH) binds to its receptor (LHR; LHCGR in human) that is a G-protein-coupled receptor (GPCR) [17]. It then activates adenylate cyclase (AC) via the heterotrimeric Gs protein, thus leading to an increase in intracellular cAMP level, that in turn activates protein kinase A (PKA) [18]. PKA regulates numerous cellular functions through phosphorylation of various specific target proteins such as cAMP responsive element binding (CREB) for genomic effects [19] or steroidogenic acute regulatory (StAR) for translocation of cholesterol into mitochondria and stimulation of steroid hormones secretion [20]. Cyclic AMP is inactivated by hydrolysis into AMP by nucleotide phosphodiesterase (PDE) [21]. In order to focus on the effects of FLX on the steps between LH binding to its receptors and adenylate cyclase stimulation, all experiments were performed in the presence of a PDE inhibitor.

Elevated levels of testosterone in α1AMPK^− ∕ −^ males are due to hyperactive Leydig cells [22] demonstrating an inhibitory action of AMPK on their steroidogenesis *in vivo*. Moreover, AMPK activation inhibits cyclic AMP-induced steroidogenesis in Leydig cell lines (MA-10 and MLTC-1 cells) by lowering the expression of key regulators of steroidogenesis like cholesterol carrier StAR and the nuclear receptor Nr4a1 [23].

In this study, we used MLTC-1 cells transiently expressing a chimeric cyclic AMP-responsive luciferase to follow real-time cAMP accumulation using oxiluciferin luminescence produced from catalyzed luciferin oxidation [24].

Considering the hypothesis of AMPK activity modulation by FLX, and the known inhibition of LH-stimulated Leydig cell steroidogenesis by AMPK, we explored whether FLX affected intracellular cAMP synthesis in MLTC-1 cell line, under hLH and/or forskolin (FSK) stimulation. Since FSK directly stimulates AC, it permits to determine more precisely the step(s) potentially affected by FLX.

The FLX effects were compared to those elicited by A-769662 and by Metformin (MET), that are direct and indirect AMPK activators respectively [13], to get a more precise view of FLX mechanism of action in Leydig cells. The data obtained clearly show that 10–100μM FLX, like MET, indirectly activates AMPK and inhibits LH-stimulated AC in MLTC-1 cells and, consequently, inhibits steroidogenesis.

## Materials and methods

### Chemicals and reagents

All chemicals were purchased from Sigma–Aldrich unless otherwise noted. Compound C, A-769662 and 1,1-dimethylbiguanide hydrochloride (Metformin; MET) were obtained from Calbiochem (Billerica, MA). A stock solution of Compound C and A-769662 were prepared in dimethylsulphoxide (DMSO) and stock solutions of MET was prepared in deionized water. Protease inhibitor cocktail was from Roche diagnostics (Mannheim, Germany). Tris/glycine buffer (10X), and Precision Plus Protein All Blue Standards (Catalog 161–0373) were obtained from Bio-Rad (Hercules, CA). Primary antibodies against AMPKα and phospho-Thr172-AMPKα were purchased from Cell Signalling technology, Inc (Danvers, MA); Anti-GAPDH (FL-335) obtained from Santa Cruz Biotechnology, INC (Texas, USA). The secondary antibody anti-rabbit IgG (H+L) (CF770 conjugated antibodies) was purchased from Biotium (Hayward, CA); FluoProbes 448 anti-Rabbit IgG antibodies and FluoProbes 546 anti-Rabbit IgG antibodies were purchased from Interchim (R.C. Montluçon-France). pGlosensor-22F cyclic AMP plasmid and CellTiter-Blue Cell viability assay (G8080) were from Promega (France), X-tremeGENE HP DNA transfection reagent was from Roche (France), recombinant hLH-C35 hormone was from Serono (Genève, Switzerland).

### Cell culture

MLTC-1 cells [25] were obtained from the American Tissue and Cell Collection (ATCC) (LGC Standards, Molsheim, France). Cells were expanded in supplemented RPMI-1640 medium (Gibco, Invitrogen, 10% fetal bovine serum, 50μg/ml gentamicin, 10 units of penicillin/ml and 10μg/ml streptomycin) and used from passes P6 to P30 as previously described [24]. All cells were grown at 37°C and 5% CO_2_.

### Plasmids, transfections

Cells (about 100,000 cells per well on a 96-well Greiner white/clear bottom plate (Dutscher, Brumath France) were transfected with pGlosensor-22F cyclic AMP plasmid using X-tremeGENE HP DNA transfection reagent. Thirty minutes before transfection, DNA (100ng plasmid per well) and X-tremeGENE HP DNA transfection reagent (0.3μl per well) were mixed together with serum-free RPMI medium. This plasmid consists in firefly luciferase sequence fused to that of the protein kinase A cyclic AMP-binding domain in a way that allows control of its enzymatic activity by cyclic AMP. The plates were then incubated overnight at 37°C under 5% CO_2_ before use of the cells in the assays.

### cAMP quantitation

Transfection supernatants were removed and replaced with medium deprived of fetal-calf serum (100μl) and containing the luciferase substrate luciferin as well as 1mM IBMX (iso-butyl-methyl-xanthine) in order to inhibit PDE activity, and thus only measure cAMP biosynhesis, i.e. only AC stimulation. The plates were incubated for 1 hour before adding FLX or AMPK modulators (A-769662, MET) at various concentrations in a 10μl volume, and then incubated for 1 hour. Finally, individual stimulating hormones or factors were added in a 10μl volume in triplicate wells to reach 700pM hLH or 10μM FSK/sub-hLH (10μM FSK together with the sub-stimulating concentration of 70pM hLH) [26]. Cyclic AMP was measured using a Polarstar Optima (BMG Labtech Sarl, Champigny sur Marne, France).

### Western-Blotting

For western-blotting experiments, total proteins were extracted from MLTC-1 cells in lysis buffer (10mM Tris, 150mM NaCl, 1mM EGTA, 1mM EDTA, 100mM sodium fluoride, 4mM sodium pyrophosphate, 2mM sodium orthovanadate, 1% Triton X-100, 0.5% NP40 containing a protease inhibitor cocktail with EDTA). Cell lysates were centrifuged at 13000g for 30 min at 4°C and the protein concentration in each supernatant was determined by a colorimetric assay (Bio-Rad DC Protein Assay; Bio-Rad, Hercules, CA). The proteins were then separated by 10% SDS-PAGE (SDS Polyacrylamide Gel Electrophoresis) and transferred onto nitrocellulose membrane (Whatman Protran, Dassel, Germany). Afterwards, the membranes were incubated in anti-AMPKα (62kDa), anti-phospho-Thr172-AMPKα (62kDa) or anti-GADPH (37kDa), diluted in 5% BSA in TBS-Tween 0.1% (final dilution 1:1000) as primary antibodies overnight at 4°C. Finally, the membranes were further incubated for 1 hour in anti-rabbit IgG (H+L) (CF 770 Conjugate) (final dilution 1:2000). The band intensity was analyzed using Odyssey Software, version 1.2 (LICOR Biosciences, Lincoln, USA). AMPKα or GADPH were used as loading controls.

### Immunocytochemistry

AMPK protein was localized in MLTC-1 cells by immunocytochemistry. MLTC-1 cells (1.10^6^ cells/ml) were washed with 1X phosphate buffered saline (PBS), fixed in paraformaldehyde (4%) for 4 min. Afterwards, cells were washed in PBS (3 x 3 min) and then permeabilized for 10 min with 0.5% Triton X-100 (Sigma-Aldrich) in PBS. Non-specific binding was blocked with PBS supplemented with 10% Donkey serum (Sigma–Aldrich) for 30 min at room temperature. Samples were then incubated overnight at 4°C with anti- AMPK diluted 1:100 in PBS-1% donkey serum. After 3 rinses with PBS, cells were incubated with FluoProbes 448 anti-Rabbit IgG antibodies (1:500 in PBS) for 1 hour at room temperature in the dark, rinsed (3 x 3 min) with PBS and incubated with 4’,6’-diamidino-2-phenylindole (DAPI) (Sigma–Aldrich) (0.05μg/ml) for 10 min. The presence of AMPK in cells was examined by confocal microscopy using a Zeiss LSM 700 confocal microscope. Negative controls were performed by omitting primary antibodies. Image analysis was performed using IMAGEJ software (http://rsbweb.nih.gov/ij/).

### MLTC-1 cells viability assessment

MLTC-1 cells were seeded in 96-well plates at 100,000 cells/well. Two days later, the medium was replaced with serum-free medium in the absence (control) or presence of FLX (0–100μM), MET (0–1000μM), or A-769662 (0–1000μM) for 1 hour at 37°C before the addition of 20μl of CellTiter-Blue Reagent (Promega, Madison, WI, USA) to each well. After having incubated for 2 hours at 37°C, changes in fluorescence were recorded with a Spectra Gemini spectrofluorimeter (Molecular Devices, Sunnyvale, CA) at an excitation wavelength of 560nm and an emission wavelength of 590nm. The fluorescent signal from the CellTiter-Blue Reagent is proportional to the number of viable cells.

### Adenosine triphosphate (ATP) concentration measurement

After the incubation of cells with or without FLX, MET, or A-769662, ATP concentration in cells was measured using the CellTiter-Glo 2.0 Assay (Promega, Madison, WI, USA). Standards were prepared from ATP standard (Promega) using serial dilutions to obtain concentrations of 1×10^−10^, 1×10^−11^ and 1×10^−12^ M. Briefly, the assay buffer and substrate were equilibrated to room temperature, and the buffer was transferred with the substrate. After 30 min, a 50μl sample of this solution was added to 50μl luciferin/luciferase reagent in 96-well white plates, the content was mixed for 2 min and incubation was continued for 10 min at room temperature. The luminescence at integration time 1000 (ms) was read using an Ascent Luminoskan Luminometer (Thermo) with PBS as a blank for each experiment.

### Primary culture of mouse testis cells

Testes were collected from adult male RjOrl:SWISS mice (Janvier Labs, France) aged 8–10 weeks, immediately after sacrifice by CO_2_-controlled asphyxia using a TEM apparatus (AETEM1). This procedure was approved by the Ethical Committee of Centre-Val de Loire region (CNRS, INRA, Universities of Tours and Orléans (France)). After removal of albuginea, testicular tissue was placed in RPMI-1640 medium. It was then chopped into small fragments using scalpel in a Petri dish containing the same medium. Afterwards the fragments were transferred in RPMI-1640 medium (4ml per testis), and gently agitated for 10 minutes at room temperature using a small magnetic bar. The dispersed cells were separated from fragments using a Pasteur pipette.

### Steroid production measurements

Progesterone levels were measured in the supernatants of cultured MLTC-1 cells and testosterone levels were measured in the supernatants of testicular Leydig cells incubated in complete RPMI medium.

For progesterone, MLTC-1 cells were first seeded in 96-well plates (100.000 cells/well) for three days and then re-suspended in serum-free RPMI and preincubated with or without FLX (100μM), MET (1mM), or A-769662 (1mM) for 1 hour. Afterwards, they were challenged for 3 hours with increasing doses of hLH alone (700pM) or Fsk/sub-hLH (10μM/70pM). Cell supernatants were collected and stored at −20°C until analysis. Progesterone production was measured with a previously described competitive ELISA assay. Briefly, a 96-wells plate was coated overnight at 4°C with a goat anti-mouse IgG antibody, 10 ng/well (UP462140, Interchim, Montluçon, France). After three washes with PBS 1X containing 0.1% Tween 20, non-specific sites were saturated 1 hour with 200μl/well of PBS-Tween 20 supplemented with 0.2% BSA. Standard progesterone (Q2600, Steraloids, USA) in PBS-Tween 20-BSA or MLTC-1 cells supernatants (25μl per well of 1:50 or 1:100 dilution) were then plated on the empty plate. Progesterone-11-Hemisuccinate-HRP (Interchim) were added, together with 36ng/well of mouse anti-P4 antibody (AbD Serotec, Biogenesis, Interchim). The plate was incubated for 4 hours at room temperature, washed and 100μl/well of TMB ELISA substrate standard solution (Interchim) was added. The mixture was incubated for 20 min at room temperature in the dark. The reaction was stopped with 2N H_2_SO_4_ and absorbance was measured at 450nm using Sunrise reader (Tecan, France).

Testosterone levels were measured by using an HTRF-based assay kit (CisBio Bioassays, Codolet, France). Briefly, primary Leydig cells in serum-free RPMI were seeded in 96-well plates at 120 000 cells/well and then incubated with or without FLX (100, 50, 25, 12.5, 6.25 μM) for 1-hour and stimulated with rhLH (5ng/10μl), or only with serum-free RPMI (control) for 3 hours at 35°C. After 3-hours of incubation at 35°C, 10μl of the culture supernatant were transferred to white 384-well microplate and 5μl of testosterone-XL665 + 5μl of anti-testosterone-Ey^3+^cryptate antibody were added. The 384-well microplate was then incubated for 1 hour at room temperature in the dark before fluorescence was measured at 620nm and 665nm after excitation at 320nm using a Mithras LB 943 plate reader (Berthold Technologies GmbH & Co. Wildbad, Germany).

### Area Under Curve (AUC) calculations and statistical analyses

The GraphPad 5 package (GraphPad Software, San Diego CA) was used for Area Under Curve (AUC) determinations of individual kinetics as well as for slope calculation by linear fitting of initial accumulation rate. Mean and SEM values for each triplicate AUCs were determined. Statistical analyses were done using one-way ANOVA followed by the Dunnett’s test posttest. For all statistical analyses, P< 0.05 was considered significant.

## Results

### Identification and localization of AMPK in MLTC-1 cell line

To consider the possible involvement of AMPK in the modulation of cyclic AMP response to LH in MLTC-1 cells, we have first checked for its presence by Western blotting and indirect immunofluorescence, using specific primary antibodies against AMPKα.

**Fig 1:**
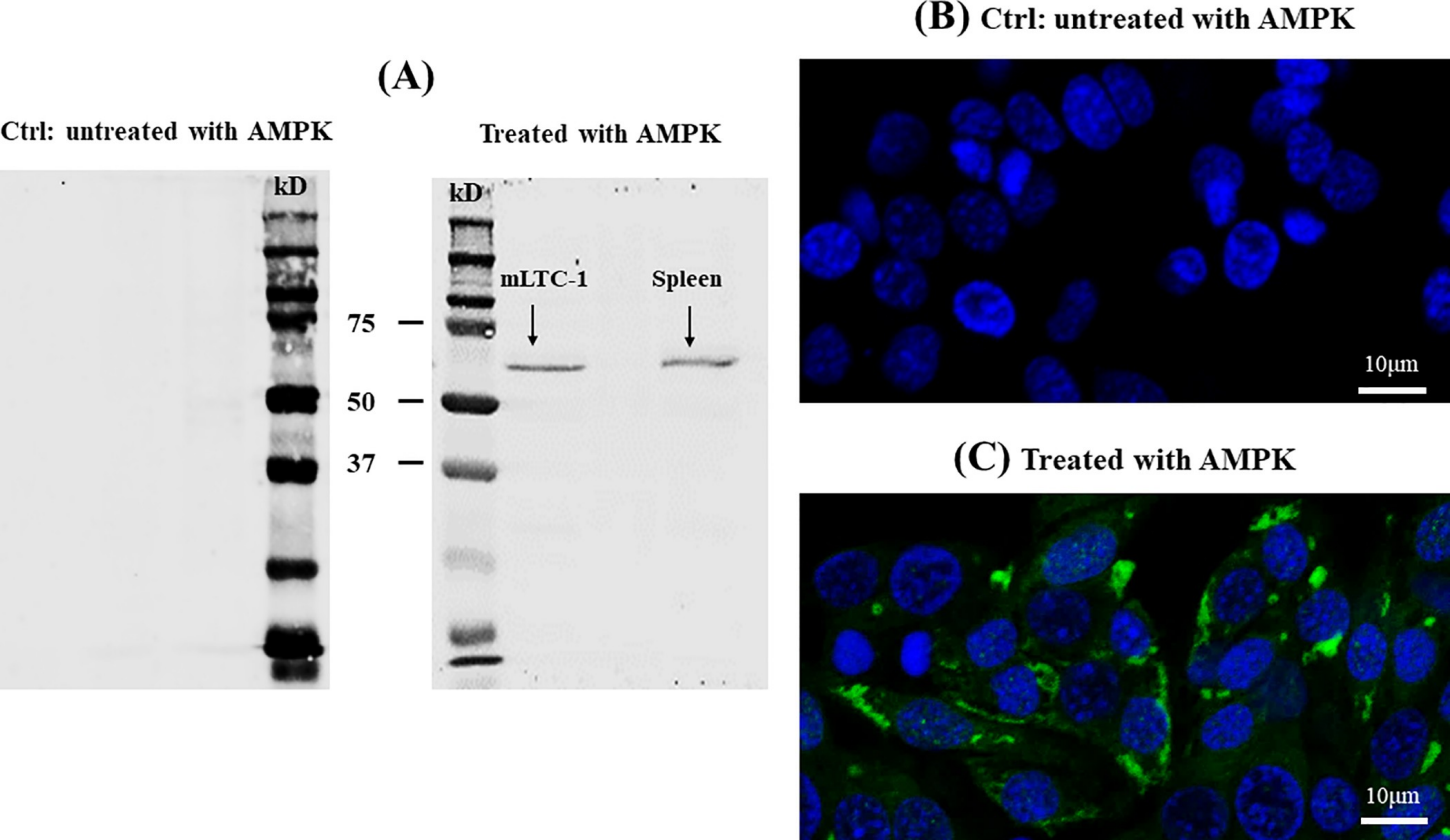
AMPK expression in MLTC-1 cells. (A) Identification of AMPK in MLTC-1. Cell lysates were resolved by SDS-PAGE, transferred to nitrocellulose membrane and then, probed with an anti-AMPKα antibody. Cell lysates from mouse spleen were used as positive controls (A: right). Negative control (Ctrl): primary antibody is missing (A: left). (B), (C) Immunolocalization of AMPK in MLTC-1 cells. Subcellular distribution of AMPK in Leydig cells was observed by confocal microscopy. AMPKα (C, green) immunoreactivity is detected in both the cell membrane and the cytoplasm. Nuclei were stained with DAPI (B, C, blue). Negative control (Ctrl): primary antibody is missing (B).

Western blotting for the catalytic α-subunit of AMPK in MLTC-1, as well as positive controls (Spleen), revealed a band with an apparent molecular weight of 62kDa for AMPKα (Fig 1A).

Immunofluorescence data show that the AMPK protein is present in MLTC-1 Leydig cells (Fig 1C), predominantly in the form of defined spots uniformly distributed in the cytoplasm. Fig 1B shows the control experiment without the primary anti-AMPKα antibody.

### Fluoxetine and AMPK modulators effects on AMPK phosphorylation in MLTC-1 cells

To assess the activation of AMPK under FLX, A-769662, or MET treatment of MLTC-1 cells, Western blot analyses were done using either an antibody against the phosphorylated/activated catalytic AMPK α-subunit. The cells were incubated in the absence or presence of FLX (6.25–100μM), A-769662 (12–1000μM), or MET (12–1000μM).

**Fig 2:**
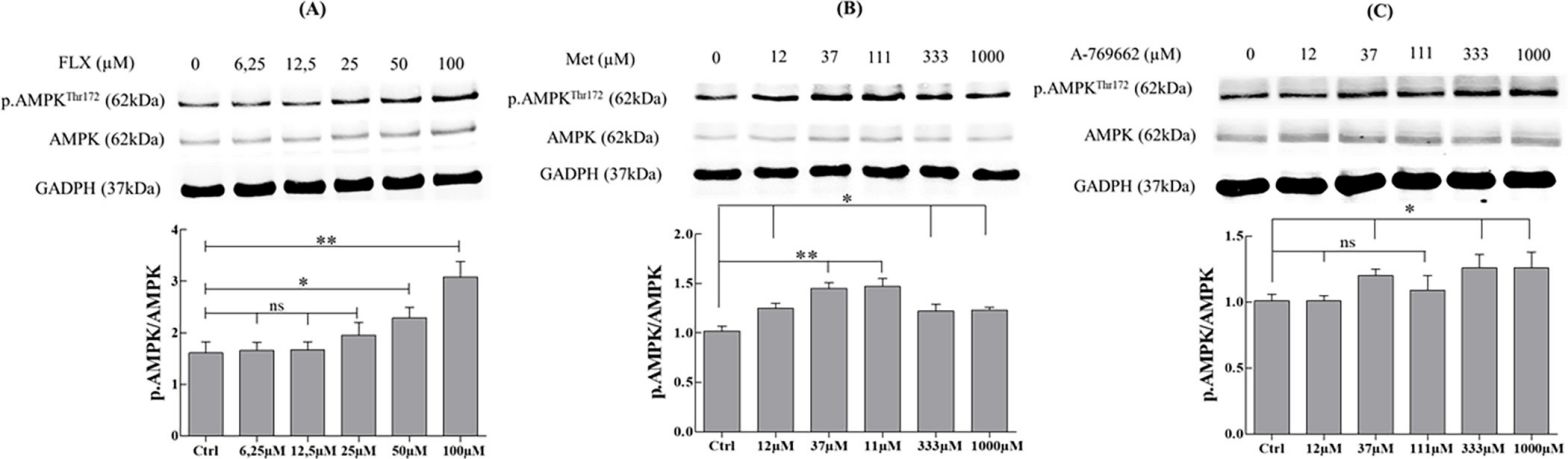
FLX enhances AMPK phosphorylation in MLTC-1 cells. After a 1-hour incubation in the presence of FLX (A), MET (B) or A-769662 (C), MLTC-1 lysates were prepared and resolved by SDS-PAGE, transferred to nitrocellulose membrane, and probed with anti-phospho-Thr172-AMPKα, anti-AMPKα and anti-GADPH antibody. Bands for phospho-Thr172-AMPKα were detected at 62 kDa (top bands). Total AMPKα (62 kDa middle bands) and GADPH (37 kDa bottom bands) were used as loading controls and the phosphorylated protein AMPKα (Thr172)/total AMPKα ratio is shown at the bottom. Data represent mean ± SEM of 6 independent experiments (n = 6). Results were analyzed by one-way ANOVA, followed by the Dunnett’s test posttest). *: significant difference (* P< 0.05; ** P<0.01), ns: no significant difference.

Fig 2A shows that FLX actually stimulates AMPK phosphorylation when present at 50μM (increase by 37.9% compared to control (Ctrl)) and 100μM (increase by 91.3% compared to Ctrl) after 60 min of incubation. MET and A-769662 also increase phospho-Thr172-AMPK levels after 60 min compared to Ctrl (Fig 2B and 2C).

### Effect of FLX and AMPK modulators on intracellular cyclic AMP response to hLH and FSK/sub-hLH

After determining the FLX and AMPK modulators concentrations that stimulate AMPK phosphorylation, we have tested these conditions to see whether they affect the LH- and FSK-stimulated cAMP accumulation in these cells. In this study, we used MLTC-1 cells transiently expressing a chimeric cyclic AMP-responsive luciferase so that real-time variations of intracellular cyclic AMP concentration could be followed using oxiluciferin luminescence produced from catalyzed luciferin oxidation [24]. The potencies of the hLH and FSK/sub-hLH were evaluated using areas under the curves (AUC) of their kinetics over 1-hour stimulations. To determine whether FLX, MET, or A-769662 affect the cAMP response to 700pM hLH (2ng/well) or to 10μM FSK + 70pM hLH (called FSK/sub-hLH as each one is at non-stimulating concentration alone), MLTC-1 cells were cultured for 1 hour in the absence (control) or presence of 0 to 100μM FLX, or of 0 to 1000μM A-769662 or MET before stimulation.

#### a) Fluoxetine

**Fig 3:**
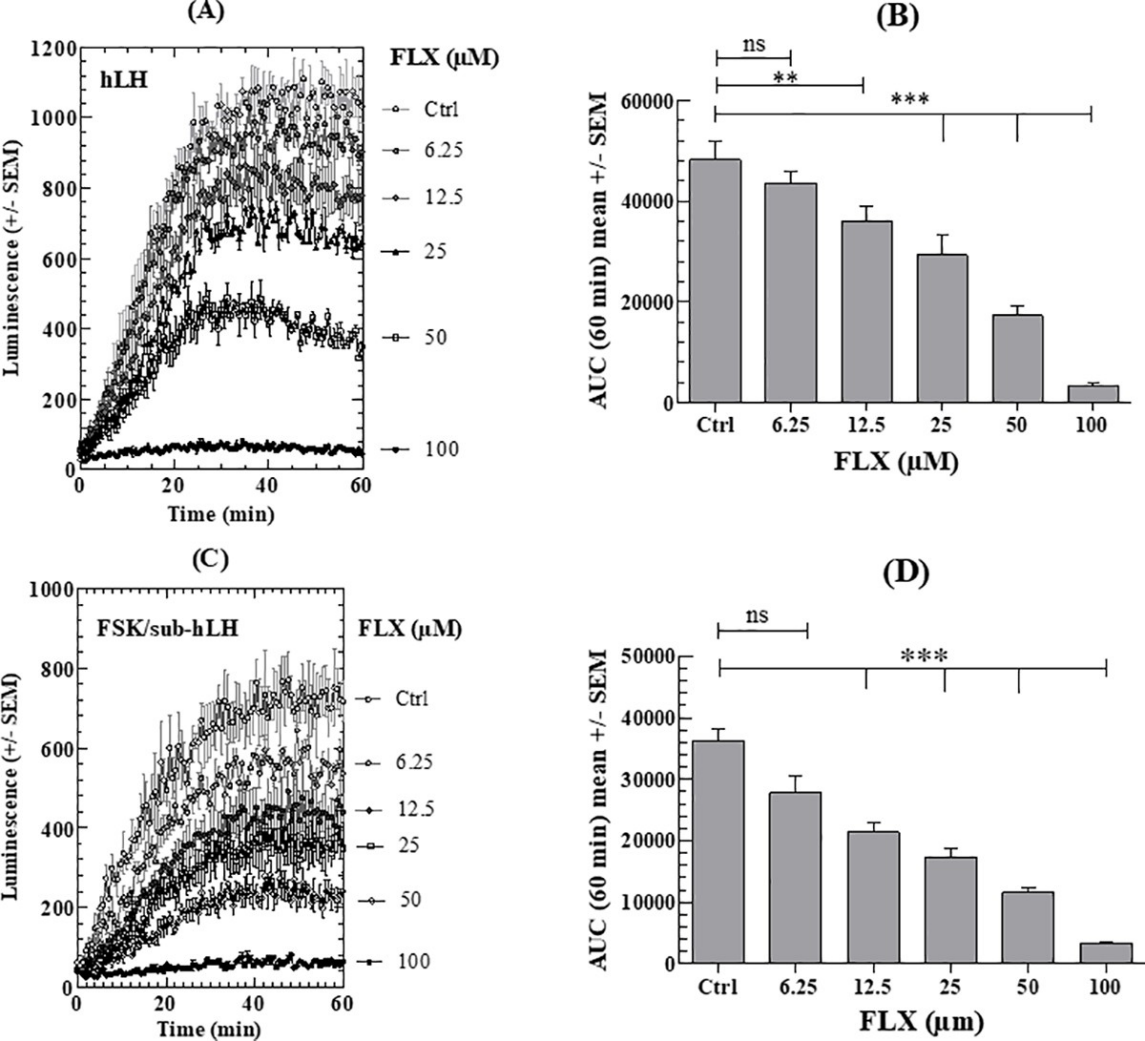
Effect of FLX on intracellular cAMP response of MLTC-1 cells to hLH or FSK/sub-hLH. (A), (C) Real-time recording of luminescence under stimulation of MLTC-1 cells by hLH and FSK/sub-hLH in the presence of 0 to 100μM FLX. (B), (D) Dose-dependent effects of FLX on hLH and FSK/sub-hLH responses respectively, determined by the Area Under Curve (AUC) of individual kinetics in Fig 3A and 3C. Data represent mean ± SEM of 3 independent experiments (n = 3). Results were analyzed by one-way ANOVA, followed by the Dunnett’s test posttest). *: significant difference (** p < 0.01; *** p < 0.001), ns: no significant difference.

The effects of FLX on intracellular cAMP in MLTC-1 cells were measured at FLX concentrations ranging between 6.25 and 100μM. Fig 3A and 3C show the fluorescence kinetics upon stimulation of transfected cAMP-dependent luciferase by hLH or FSK/sub-hLH respectively. Clear significant FLX dose-dependent decreases of LH- and FSK/sub-LH responses are observed as calculated by the areas under the curves (AUC) (decrease by 25.4–40.7% at 12,5μM; by 39.3–51.9% at 25μM; by 64.2–68% at 50μM compared to Ctrl) (Fig 3B and 3D respectively). At 100μM concentration, FLX almost completely abolishes the LH- or FSK/sub-hLH-stimulated cAMP accumulation responses.

#### b) A-769662

**Fig 4:**
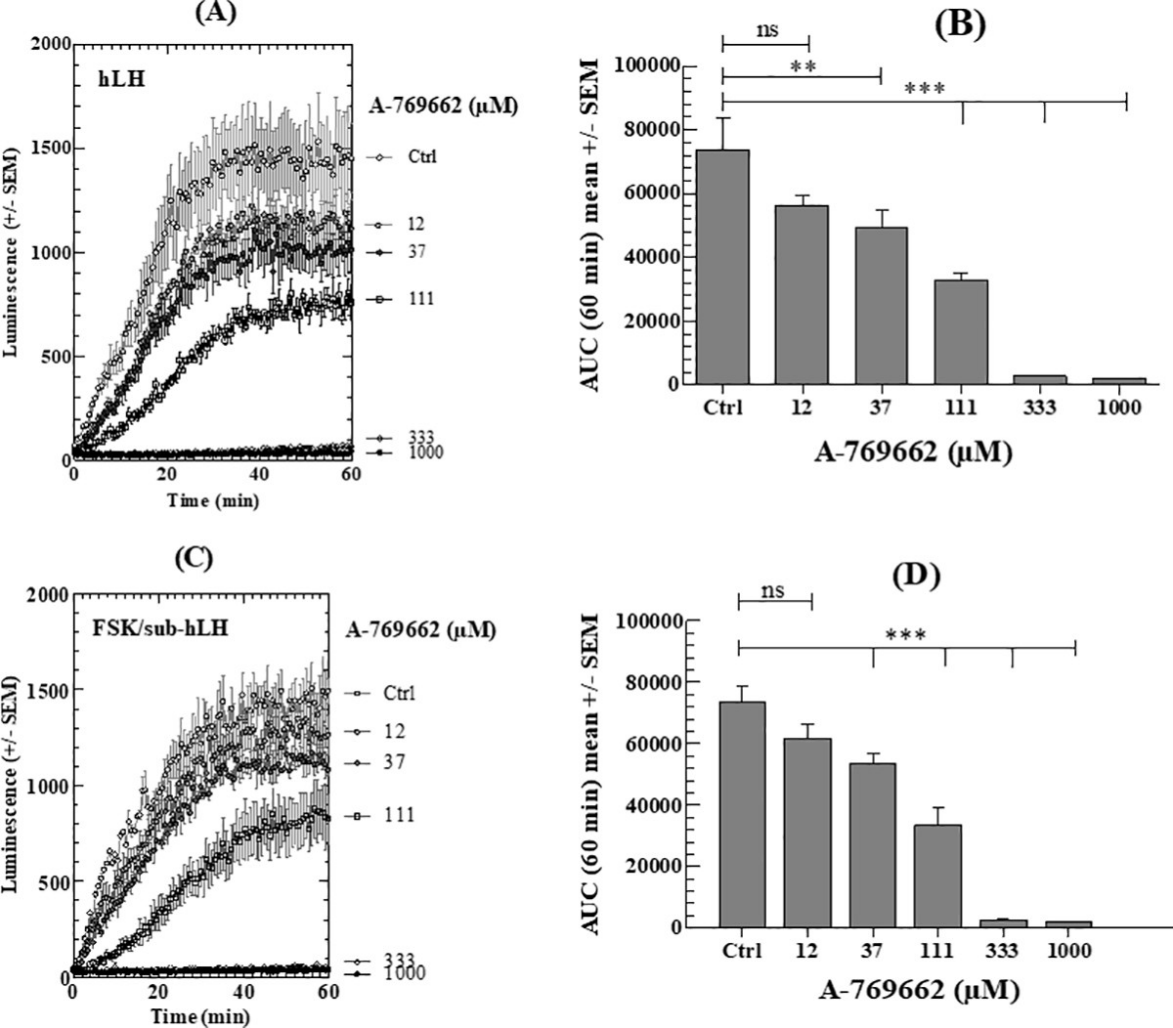
Effect of A-769662 on intracellular cAMP response of MLTC-1 cells to hLH or FSK/sub-hLH. (A), (C) Real-time recording of luminescence under stimulation of MLTC-1 cells by hLH and FSK/sub-hLH respectively in presence of 0 to 1000μM A-769662. (B), (D) Dose-dependent effects of A-769662 on hLH and FSK/sub-hLH responses respectively, determined by the Area Under Curve (AUC) of individual kinetics in Fig 4A and 4C. Data represent mean ± SEM of 3 independent experiments (n = 3). Results were analyzed by one-way ANOVA, followed by the Dunnett’s test posttest). *: significant difference (** p < 0.01; *** p < 0.001), ns: no significant difference.

The direct AMPK stimulator A-769662 also induces a complete dose-dependent decrease in the intracellular cAMP accumulation kinetics to hLH or FSK/sub-hLH (Fig 4A and 4C). These decreases are highly significant as calculated by these kinetics AUCs (decrease by 27–32.8% at 37μM; by 55% at 111μM compared to Ctrl) (Fig 4B and 4D). At 333 and 1000μM of A-769662, there is a complete abolition of the LH- and FSK/sub-hLH promoted cAMP responses.

#### c) Metformin

**Fig 5:**
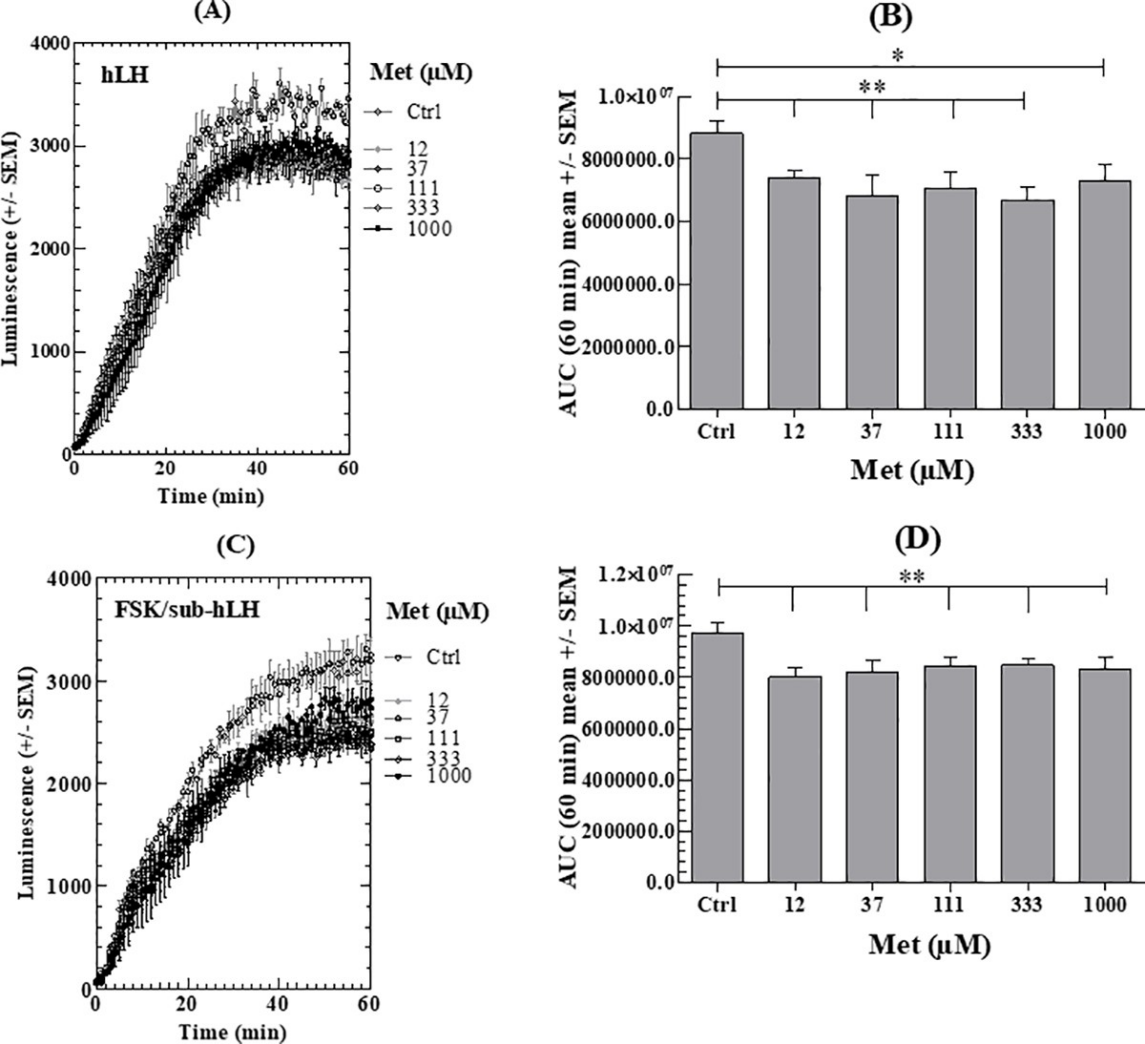
Effect of MET on cAMP response of MLTC-1 cells to hLH or FSK/sub-hLH. (A), (C) Real-time recording of luminescence under stimulation of MLTC-1 cells by hLH or FSK/sub-hLH in the presence of 0 to 1000μM MET. (B), (D) Dose-dependent effects of MET determined on hLH and FSK/sub-hLH responses respectively, by the Area Under Curve (AUC) of individual kinetics in Fig 5A and 5C. Data represent mean ± SEM of 3 independent experiments (n = 3). Results were analyzed by one-way ANOVA, followed by the Dunnett’s test posttest). *: significant difference (* P< 0.05; ** P<0.01).

MET, in contrast to A-769662, indirectly activates AMPK. Fig 5 shows that, also in contrast to A-769662, it only provokes a partial inhibition in the subsequent cAMP response to hLH or to FSK/sub-hLH at all the concentrations tested between 12 and 1000μM (about 20% compared to Ctrl).

#### d) Compound C

**Fig 6:**
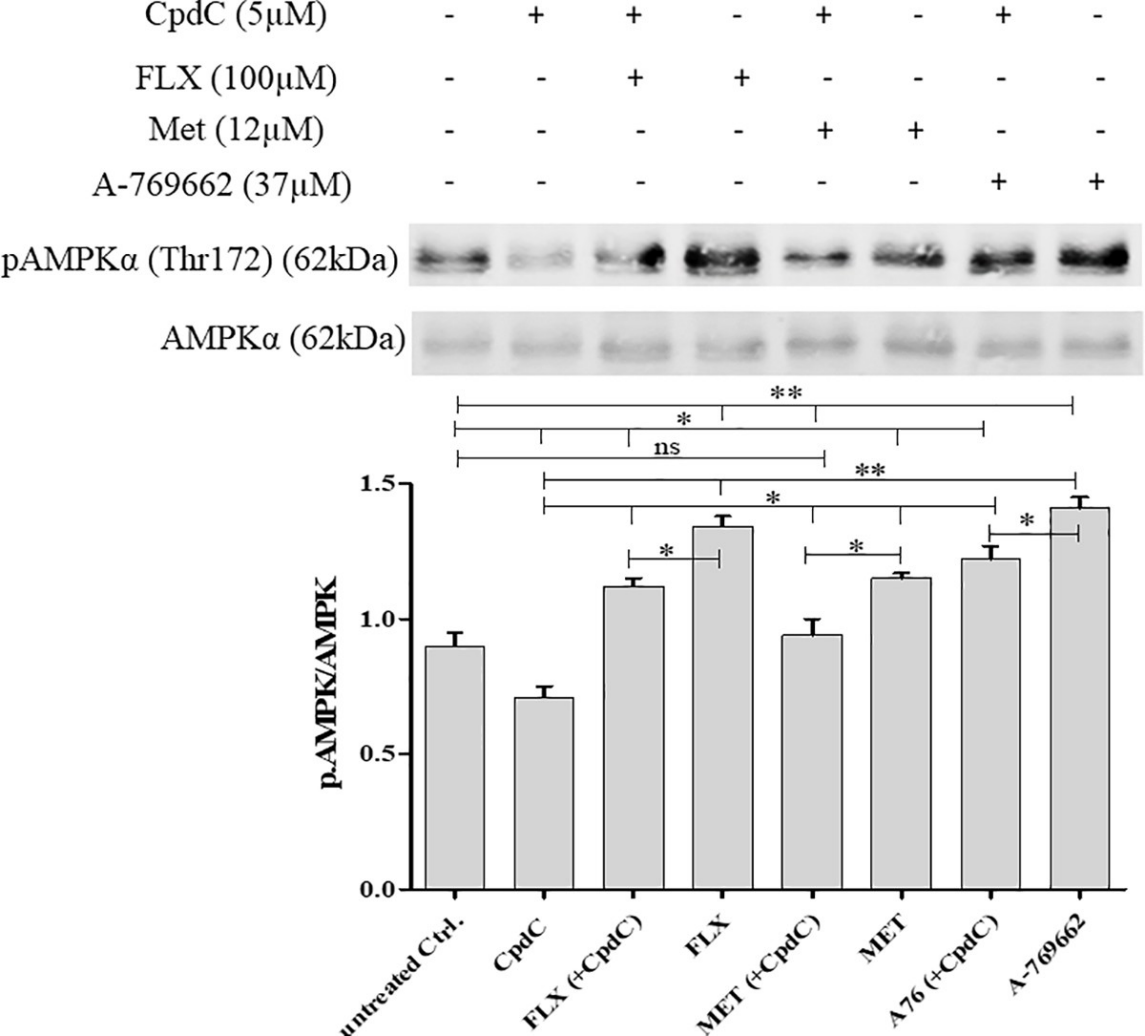
Effect of Compound C on FLX, MET, or A-769662 induced AMPK phosphorylation. MLTC-1 cells were preincubated with or without compound C (5μM) for 30 min before the addition of 100μM FLX or 12μM MET or 37μM A-769662, and incubated for an additional 60 min. The experiment was performed 4 times independent experiments (n = 4). Ratio values are means ± SEM. Results were analyzed by one-way ANOVA, followed by the Dunnett’s test posttest). *: significant difference (* P< 0.05; ** P<0.01), ns: no significant difference.

**Fig 7:**
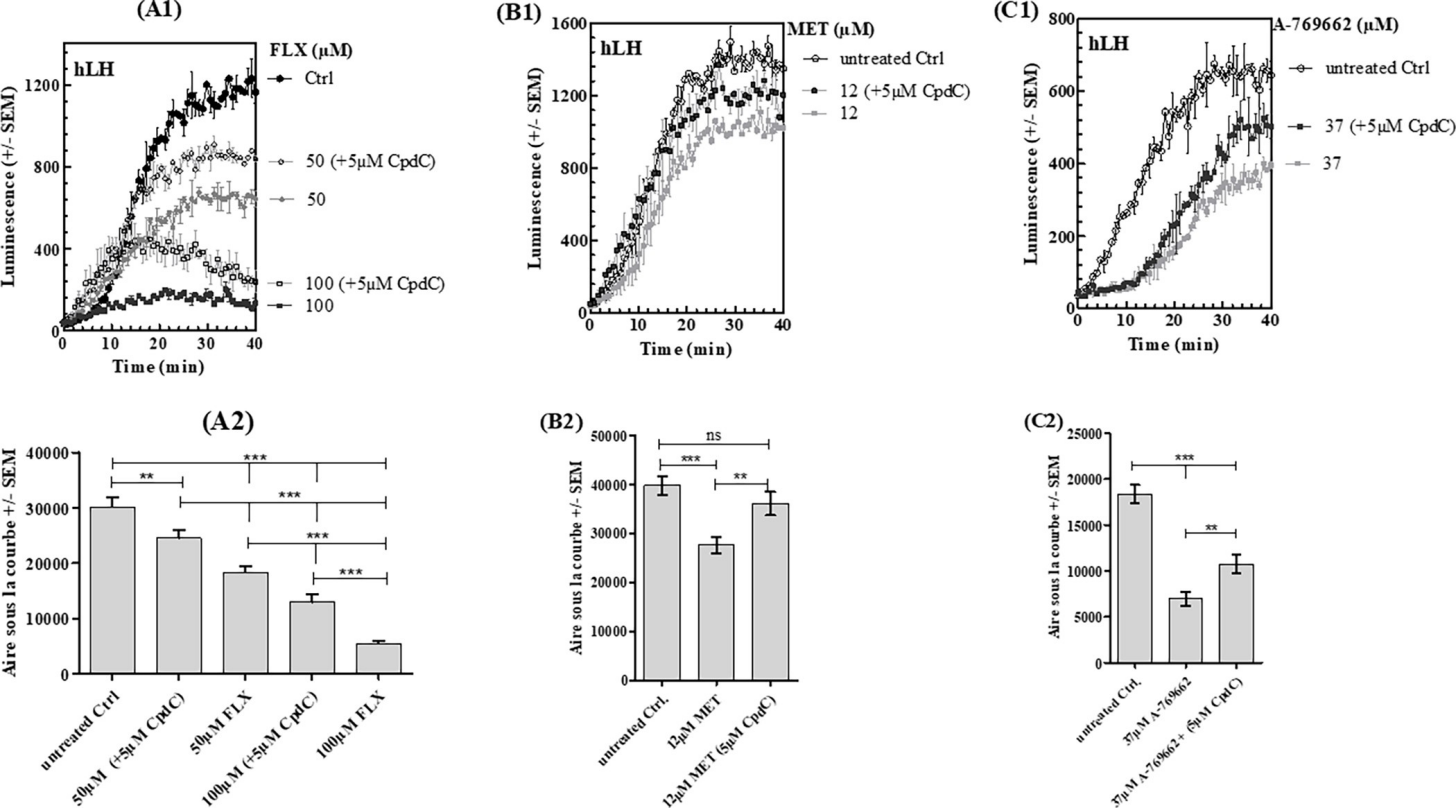
Effect of Compound C on FLX, MET, or A-769662 on intracellular cAMP response of MLTC-1 cells to hLH. (A1), (B1), (C1) Real-time recording of luminescence under stimulation of MLTC-1 cells by hLH in presence of FLX, MET, or A-769662. (A2), (B2), (C2) Dose-dependent response to FLX, MET, or A-769662 determined by the Area Under Curve (AUC) of individual kinetics in figs A1, B1 and C1 respectively. Data represent mean ± SEM of 3 independent experiments (n = 3). Results were analyzed by one-way ANOVA, followed by the Dunnett’s test posttest). *: significant difference (** p< 0.01; *** P<0.001), ns: no significant difference.

In order to ascertain that the observed changes were actually due to AMPK activation, we used the AMPK inhibitor CpdC to check that it could counteract their effect. MLTC-1 cells were pre-treated with 5μM CpdC for 30 min, and then treated with MET (12μM) A-769662 (37μM) or FLX (100μM) for 60 min. The increases in AMPK phosphorylation by FLX, MET or A-769662 were significantly attenuated in the presence of CpdC (Fig 6). In parallel, cyclic AMP responses to hLH and FSK/sub-hLH in MLTC-1 cells were partially restored (Fig 7).

### Effect of FLX and AMPK modulators on MLTC-1 viability and ATP concentration

**Fig 8:**
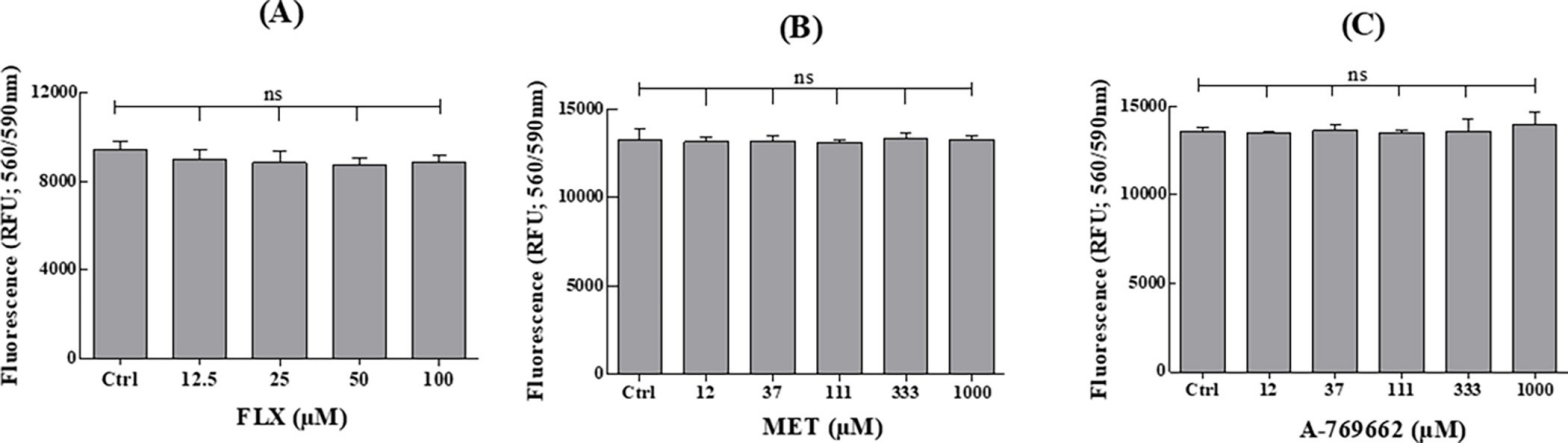
Effect of FLX, MET, or A-769662 on MLTC-1 cells viability. Cells were incubated at 37°C for 1 hour in the presence or absence of FLX (A) or MET (B) or A-769662 (C). The experiments were repeated 6 times, values (%) are mean ± SEM (n = 6). Results were analyzed by one-way ANOVA, followed by the Dunnett’s test posttest). ns = no significant difference.

We then checked that the observed effects were not due to losses in cell viability or intracellular ATP availability. After 1-hour of incubation in the presence of FLX (6.25–100μM), MET (12–1000μM), or A-769662 (12–1000μM) and 2-hours of preincubation with CellTiter-Glo 2.0 Assay, cell viability was found to be unaffected when compared to control (Fig 8).

**Fig 9:**
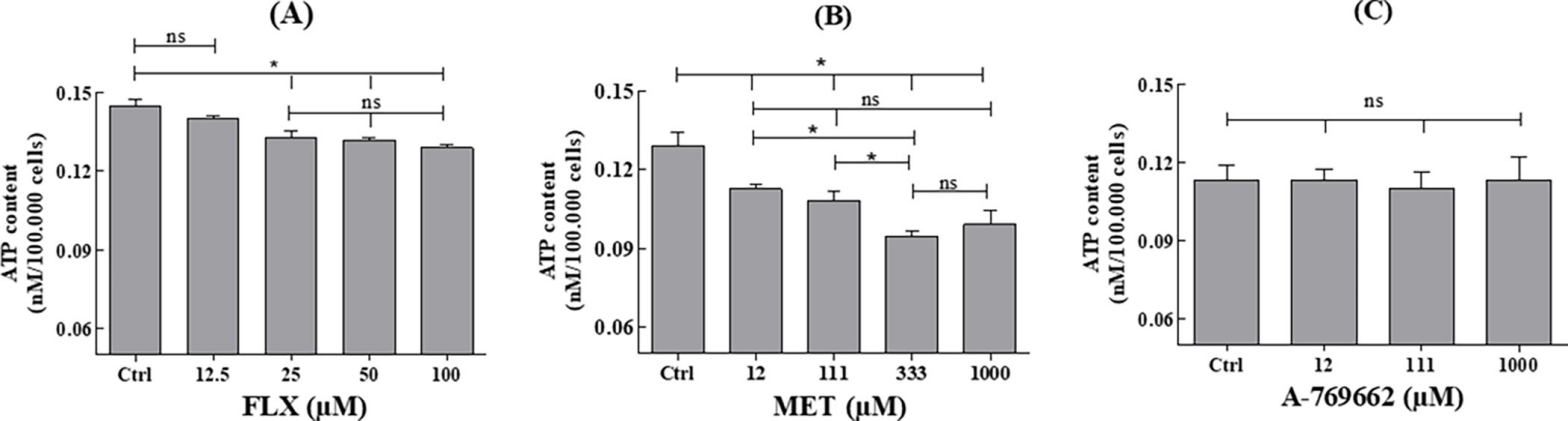
Effect of FLX or MET or A-769662 on ATP concentration in MLTC-1 cells. ATP concentration in living cells was monitored using the Cell-Titer-Glo Assay. Cells were incubated at 37°C in the presence or absence of FLX (A) or MET (B) or A-769662 (C). The experiments were repeated 6 times independent experiments, values (%) are mean ± SEM (n = 6). Results were analyzed by one-way ANOVA, followed by the Dunnett’s test posttest). *: significant difference (* P< 0.05), ns: no significant difference.

The intracellular ATP concentration was measured after incubation of MLTC-1 cells with FLX using the Cell-Titer-Glo Assay. Fig 9A shows that FLX significantly decreases intracellular ATP concentration only at 25μM or more. We also observed that MET, but not A-769662, induces a significant decrease in intracellular ATP concentration (Fig 9B and 9C).

### FLX and AMPK modulators inhibit hLH or FSK/sub-hLH-induced steroidogenesis in MLTC-1 cells and testicular Leydig cells

**Fig 10:**
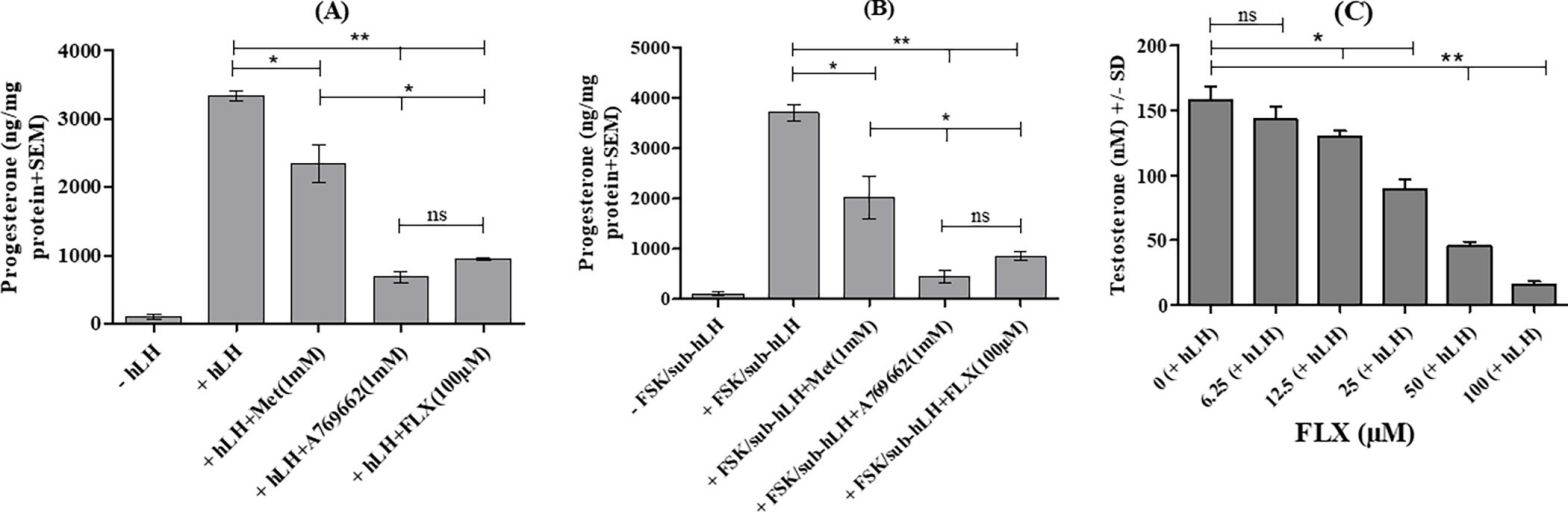
Effect of fluoxetine in hLH or FSK/sub-hLH -promoted steroid production in MLTC-1 and primary Leydig cells. (A), (B) MLTC-1 cells were preincubated with or without FLX (or MET, or A-769662) for 1 hour and then the cells were stimulated for 3 hours with hLH or FSK/sub-hLH before progesterone productions were measured. (C) Primary Leydig cells were preincubated with or without FLX for 1 hour and then the cells were stimulated for 3 hours with hLH before testosterone productions were measured. Data are means ± SEM of 3 independent experiments performed in duplicate (n = 3). Results were analyzed by one-way ANOVA, followed by the Dunnett’s test posttest). *: significant difference (* P< 0.05; ** P<0.01), ns: no significant difference.

Finally, the physiological significance of intracellular cAMP modulations by FLX and of AMPK modulators was further studied by looking at the specific steroid secretion of MLTC-1 cells (progesterone) and testicular Leydig cells (testosterone). After 1 hour of incubation with either FLX, A-769662, or MET, and 3 hours of stimulation by hLH (e.g.: the product is present for 4 hours in total during the assay of steroidogenesis), we observed that the increase of progesterone production by hLH or FSK/sub-hLH in MLTC-1 cells was inhibited in the presence of FLX, or MET or A-769662 (Fig 10A and 10B). We also found that the decreases of progesterone production caused by FLX and A-769662 were stronger than with MET.

We also observed that FLX reduced testosterone production under hLH stimulation, in primary testicular Leydig cells (Fig 10C).

## Discussion

The present report shows that the antidepressant fluoxetine (FLX), at μM concentrations, exerts a strong inhibition on LH-stimulated cyclic AMP synthesis and progesterone secretion in MLTC-1 cells, at least partly mediated by AMPK activation.

Like the AMPK activators A-769662 and MET, FLX is shown here to increase AMPK phosphorylation, to decrease the cAMP response to hLH or FSK, and also to reduce the progesterone and testosterone secretions under hLH or FSK/sub-hLH stimulation in MLTC-1 cells and also in primary testicular mouse Leydig cells. AMPK phosphorylation at threonine 172 of its catalytic α subunit by protein kinases such as Liver kinase B1 (LKB1) or Calcium/calmodulin-dependent protein kinase kinase β (CaMKK), triggers its activation. It is the AMP-induced conformational change in AMPK structure that allows its phosphorylation by these kinases [11].

FLX at 10–100μM concentration exerts a dose-dependent inhibitory effect on the cAMP response of MLTC-1 cells to hLH. In order to identify more precisely the AMPK target in the hLH to cAMP pathway, we also tested the effects of FLX and AMPK modulators, on the cAMP response to the direct AC activator FSK. In a previous report [26], 10μM FSK alone was shown unable to stimulate cAMP accumulation in MLTC cells. In contrast, 10μM FSK together with a sub-stimulating hLH concentration (70pM), promoted a full cAMP response [26]. Therefore, the FSK/sub-hLH mix was used to study the effects of FLX and AMPK modulators on direct AC stimulation. Strong inhibition by FLX was observed when it was introduced one hour before 700pM hLH or 10μM FSK/sub-hLH, but was also significant when introduced at the same time as hormone and/or FSK at T0. At 100μM FLX the hLH and FSK/sub-hLH responses are almost totally abolished even when FLX was introduced at the same time as the hormone or FSK.

The rapid inhibition by FLX of both cAMP synthesis and progesterone secretion in MLTC-1 stimulated with hLH or FSK/sub-hLH, leads to the hypothesis that FLX affects the LHR-G_s_-AC-cAMP-PKA-StAR cascade [27]. We checked whether it was also the case in primary cells from adult mice testes, and observed that FLX, indeed reduced testosterone production under stimulation with hLH. It is highly probable that this inhibition of steroidogenesis is due to inhibition of cyclic AMP accumulation, as shown in MLTC cells. What mechanism(s) cause(s) the observed quick decrease in cyclic AMP response to LH in the presence of FLX?

The ATP concentration decrease in MLTC-1 cells after a 1-hour treatment with 25–100μM FLX, in agreement with previous data [9] suggesting that FLX partially decreases ATP synthesis by reducing respiratory chain activity. FLX is principally a SSRI but it has also been shown to possess a wide range of biological activities such as interaction with Na^+^ and K^+^ and Ca^2+^ channels [4, 7]. In mitochondria, FLX indirectly affects electron transport and (F1F0) ATPase activity, and thus inhibits oxidative phosphorylation [28]. These FLX effects on mitochondrial activities may also result from interaction with Voltage-dependent anion channels (VDAC) and conductance decreasing of a Ca^2+^ channel [29] adenine nucleotides [30], other metabolites [31–32], as well as preventing PTP opening by preventing the release of accumulated Ca^2+^ and by swelling of energized mitochondria and inhibiting release of cytochrome C from mitochondria [6].

To confirm AMPK activity involvement in FLX effects on cAMP response to LH, CpdC was used [33]. Indeed, AMPK inhibition by CpdC completely impaired FLX inhibitory effect on cAMP response to hLH. Further support for FLX effects through AMPK activation was that the AMPK activators MET and A-769662, like FLX, increased AMPK phosphorylation and decreased cAMP synthesis rate under hLH or FSK stimulation. In addition, the AMPK inhibitor CpdC prevented the decreased cAMP response to hLH in MLTC-1 cells caused by A-769662, MET, or FLX.

FLX might nevertheless also act at low concentrations through another pathway, independent of AMPK. Indeed, no increase in AMPK phosphorylation was observed at 12.5 μM and 25μM FLX concentrations, although these did reduce cAMP accumulation under LH stimulation. FLX at low concentrations could directly inhibit adenylate cyclase activity through depletion of its substrate ATP.

MET is biguanide, synthetic derivatives of guanidine, which is now generally the first-choice drug for treatment of type 2 diabetes. As a cation, MET accumulates in mitochondria because of the electrical gradient of the inner membrane and inhibits Complex I of the mitochondrial respiratory chain [34]. Its uptake into many cells, like hepatocytes, is fostered by the organic cation transporter OCT1 despite having poor plasma membrane permeability [35]. MET-dependent inhibition of oxidative phosphorylation (OXPHOS) promotes an increase in intracellular ADP and AMP, and leads to indirect AMPK activation [34, 36] without affecting cell viability. Surprisingly, we have found that there was no further decrease in cAMP response to hLH or FSK at MET concentrations above 12μM. Decreased ATP level and increased active AMPK phosphorylation were both observed in the same range of MET concentrations, confirming that AMPK activation was indeed caused by diminished ATP concentration. Evidence in favor of an effect of MET on AMP through an AMPK-independent mechanism was reported recently. Indeed, MET has been shown to inhibit AMP deaminase activity, an enzyme degrading AMP, and this would lead to increased AMP concentration [37], leading to direct adenylate cyclase inhibition, and consequently to decreased cAMP accumulation [38]. In any case, the two mechanisms (AMPK-dependent and AMPK-independent) are mutually non-exclusive and possibly complementary to explain the decreased cAMP response to hLH or FSK/sub-hLH in MLTC in presence of MET.

Unlike MET, A-79662 is a direct AMPK activator [37–39] and is shown here to repress cAMP synthesis induced by LH or FSK/sub-hLH in MLTC-1 cells. This repression is complete only at 333 and 1000μM but in the same range as that provoking full AMPK activation in MLTC cells. Furthermore, A-769662 has no effect on ATP concentration, in full agreement with A-769662 being a direct activator of AMPK in contrast to biguanides including MET [38–39].

Interestingly, A-769662 concentrations higher than 50μM increase extracellular ATP (eATP) levels and enhance intracellular calcium levels in astrocytes [40]. It is known that eATP transduces purinergic signal [41–42]. It can be speculated that the abolition of the intracellular cAMP response to LH or FSK/sub-hLH by A-769662 might be partly due to this A-769662 side effect, through extracellular ATP. We actually did not observe any significant variation in ATP concentration at high A-769662 concentrations, indicating that its reduction of the cAMP response to LH or FSK stimulation is primarily exerted through an AMPK-dependent mechanism.

**Fig 11:**
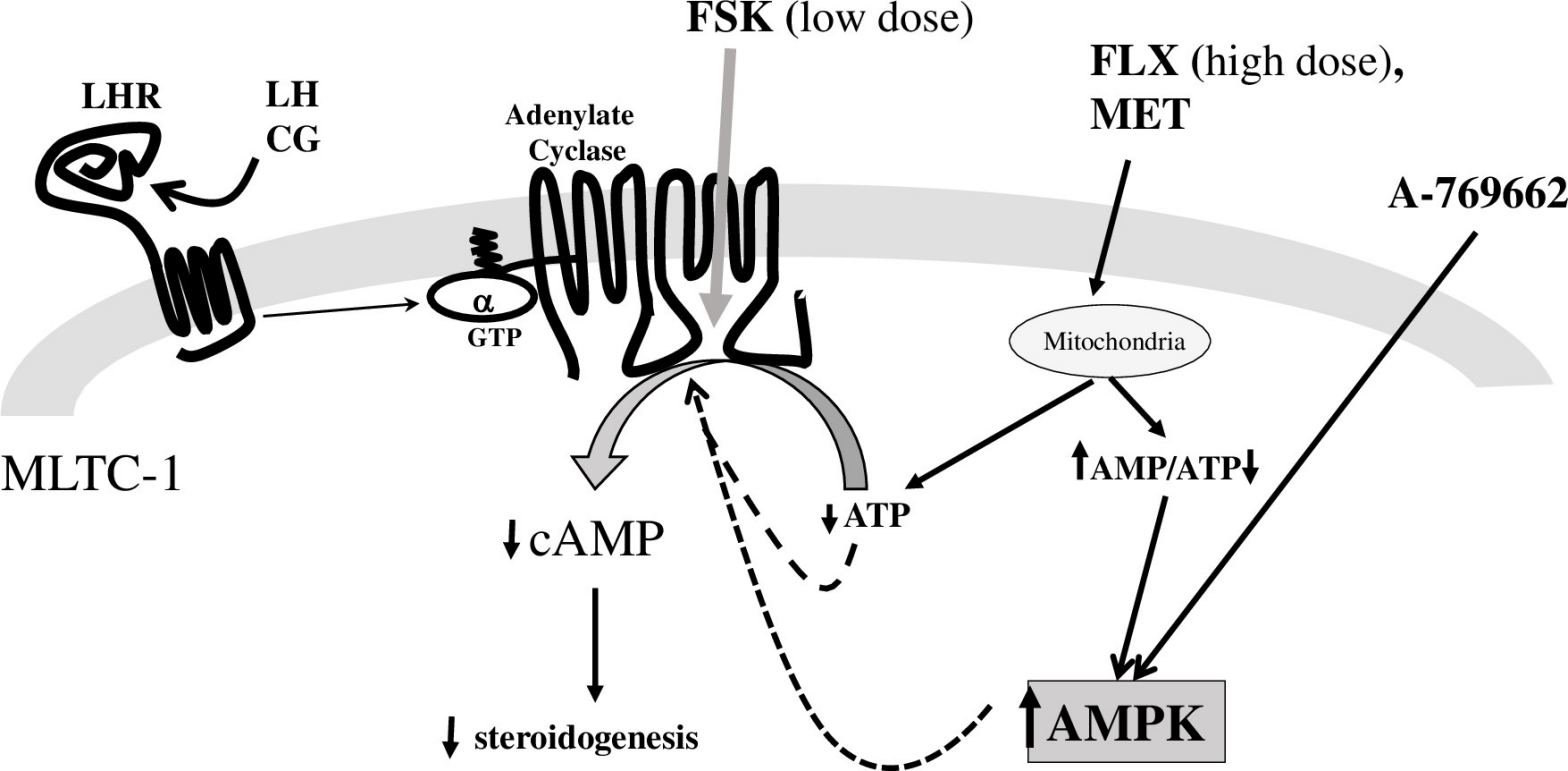
Proposed mechanisms of FLX inhibition of cAMP accumulation in MLTC-1 cells under hLH stimulation. Up or down arrows next to a metabolite or a protein show how the concentration or the activity changes in response to FLX, A-769662, or MET. After uptake into MLTC-1 cells MET and FLX accumulate in mitochondria where they inhibit the respiratory chain, lowering cytoplasmic ATP and increasing ADP and AMP. Increase of the AMP/ATP ratio activates AMPK, which in some way inhibits AC activity. Moreover, the decrease in ATP (AC substrate) also slowers cAMP synthesis. Unlike MET and FLX, treatment with A-769662 activates AMPK without inhibiting cellular ATP levels. As a consequence, the effect on the LH-stimulated cAMP pathway is decreased in MLTC-1 cells incubated with FLX, A-769662 or MET, the effect being AMPK-dependent and/or AMPK-independent; dashed black arrows mark are used for hypotheses.

Based on the data presented here, we propose a model with a central role for AMPK on the inhibition of adenylate cyclase activity in response to FLX, MET and A-769662 in MLTC-1 cells (Fig 11). In this model, MET and FLX both activate AMPK indirectly, probably by decreasing cellular energy levels through the inhibition of mitochondrial respiration thus leading to an increase in AMP+ADP/ATP ratio, whereas A-769662 directly activates AMPK. Consequently, AMPK activation induces a decrease in the cAMP synthesis catalyzed by adenylate cyclase under LH or FSK stimulation. It is interesting to note, however, that MET and FLX could directly decrease cAMP production independently of AMPK, maybe, for example, by decreasing intracellular ATP levels.

The simplest mechanism would be that AMPK directly phosphorylates adenylate cyclase on specific serine and/or threonine residues, leading to a diminution of its activity. Previous works have described AC activity control through its phosphorylation by PKA [43–44] but not yet, to our knowledge, by AMPK.

AMPK has been identified as a regulator of numerous ion transport proteins [45–49] which could affect plasma membrane potential and consequently impact AC activity. AMPK has also been shown to stimulate PDE activity leading to decrease in cAMP levels in certain cells [50]. However, since the experiments were performed in the presence of the PDE inhibitor IBMX, this hypothesis cannot be retained to explain our data. Moreover, in experiments performed in the absence of IBMX (not shown), we obtained the same results as in its presence, ruling out a significant short-term effect of AMPK on PDE activity in MLTC-1 cells. In addition to this, mTOR is a known functionally important downstream target of AMPK [51–52] that could inhibit cAMP synthesis [53]. These hypotheses need further research to establish the precise mechanism(s) by which FLX affect AMPK and adenylate cyclase activities.

The AC-cAMP-PKA cascade has been shown to be linked to the pathogenesis and treatment of depression [27]. Modulation of activity of CREB and its cofactors [54] at its target genes causes the cellular adaptations underlying the antidepressant actions of FLX [55–56]. For the time being, it is not clear whether the blood testosterone level of FLX-treated depressed male patients is reduced [57] or not [58]. If Leydig cells cannot produce enough testosterone, spermatogenesis fails [59–60] and males become oligospermic and exhibit reduced fertility. Nevertheless, the clinical observations were done in patients after long-term FLX treatment (3–12 months) and might be unrelated to the acute mechanisms described in the present report.

**In conclusion**, we have identified in MLTC-1 cells, an active repression mechanism by FLX involving AMPK as demonstrated by the similar effects exerted by MET and A-769662. As FLX was found to inhibit steroidogenesis in both MLTC-1 and testicular Leydig cells, we think that it should provide novel lines of investigation concerning long-term mechanisms by which steroidogenesis might be impaired in depressed patients treated with fluoxetine, or in diabetic patients treated with metformin.
